# Editorial: Wearable Robots and Sensorimotor Interfaces: Augmentation, Rehabilitation, Assistance or Substitution of Human Sensorimotor Function

**DOI:** 10.3389/fnbot.2022.954865

**Published:** 2022-06-23

**Authors:** Chad G. Rose, Dongming Gan, Irfan Hussain

**Affiliations:** ^1^Department of Mechanical Engineering, Auburn University, Auburn, AL, United States; ^2^Polytechnic Institute, Purdue University, West Lafayette, IN, United States; ^3^Department of Mechanical Engineering, Center of Autonomous Robotics Systems, Khalifa University, Abu Dhabi, United Arab Emirates

**Keywords:** wearable robotics, prosthetics, control, electroencephalography, electromyography, neurorehabilitation, user-centered design

Research in wearables for rehabilitation, assistance, and augmentation have generally focused solely on motor or sensory (in particular haptic) aspects. Considering the combined sensorimotor aspects of such wearables can create new research directions, and stands to improve the function seen in state of the art devices. In this topic, authors contributed works along three broad themes: design, control, and assessment.

With the design theme of the topic, a major motivation and emphasis was placed by Varghese et al. and Alvarado-Rivera et al. on the importance of managing the pressures at the interface between the wearer and the robot, or the combined wearer-robot system and the environment in order to achieve high performance goals. The other papers in this group focused on the challenging design requirements in prosthetic hands, proposing designs to incorporate human-like capabilities or distribute the control strategies into passive or mechanically-intelligent structures. Gao et al. presented a differential mechanism to accommodate the high degrees of freedom of the hands with a reduced set of actuators. Hocaoglu and Patoglu focused on recreating the variable stiffness capabilities of human hands with a novel mechanism. Lastly, Weiner et al. developed a prosthetic hand capable of semi-autonomous grasping, relying on a multi-modal sensor network combined with adaptive underactuated mechanisms.

Several papers within the topic coalesced around a theme of control and intent detection to support wearable robotic implementations. Gantenbein et al. presented a review of intent detection strategies for upper limb orthoses. Hocaoglu and Patoglu presented an sEMG-based control strategy to leverage the performance of the variable stiffness actuator introduced in a previous paper in the topic. Instead of seeking to recreate the human impedances, Kumar et al. proposed an admittance controller to enable human-like gait on arbitrary slopes. Also aiming to improve the control of lower limb prosthetics, Hong et al. presented the connections between torso kinematics and gait phase estimation. Lastly, with a focus on rehabiliation instead of augmentation or prosthetics, Topini et al. proposed an admittance controller for use in VR training environments, and examined its performance in a single subject pilot.

Several manuscripts focused on a third theme, that of assessment of the combined human-robot system. Dissanayake et al. investigated the fatigue in upper limb motions via changes detectable via EEG. Lastly, Patrick, Kumar, and Hur and Patrick, Kumar, Hong, and Hur examined the biomechanical implications of the orthotic and prosthetic kinematic structure, respectively, on the kinematics and kinetics of gait.

Taken together, the works in this Research Topic underscore the far ranging applications of considering sensorimotor aspects in wearable robotics, ranging from the design of human-robot interfaces to EEG assessments. These new designs and results are another step toward achieving the potential of wearables, but there are still many open questions and unknowns in this highly interdisciplinary field, which will require further investigation and collaboration.

A fundamental limitation in the current research model is the difficulty at achieving long duration studies with large population sizes. In some areas, this may be overcome via commercialization, but in others, the field may need to rely on large studies on standardized equipment, such as open-source designs, such as the Open Source Leg (Azocar et al., 2020), which can be a starting point toward accumulating the “big data” which drives much innovation in robotics and machine learning.

To make the next generation of devices, controllers, and interfaces, assessment and inclusion of end users in the initial design and validation process, such as the usability and evaluation from authors such as Gantenbien et al. are the first steps in this direction. Next steps may look like the creation of standardized performance metrics and methods, such as those proposed for prosthetics (Light et al., 2002) for orthoses, or open-source designs for wearer surrogates such as mannikins to complement standard object sets (Calli et al., 2015). Additional efforts aimed at enabling end users to be not only the assessors, but the designers, can further democratize and accelerate the design process.

While kinematic and kinetic assessments have been well established, with advances presented in this topic, the field can also benefit from additional investigation into the connections between sensory and motor function, such as Lowrey et al. (2020). Future work could further tease out the interconnections between motor and sensory function, identifying new design guidelines, control strategies, and assessment methods for all the wearable devices including the one newly proposed and known as supernumerary robotics limbs (Hussain and Prattichizzo, 2020).

Lastly, and at the risk of understatement, the future work is dependent on continued advancement in the miniaturization, proliferation, and optimization of the requisite mechatronic subsystems. As these fields advance, wearable robotics need to be prepared to take advantage of the opportunities the latest, lightest, most efficient, and lowest-cost batteries, sensors, and actuators, and computational resources affords us.

## Author Contributions

All authors listed have made a substantial, direct, and intellectual contribution to the work and approved it for publication.

## Funding

This work was partially supported by the Khalifa University of Science and Technology under Award No. RC1-2018-KUCARS and FSU-2021-019.

## Conflict of Interest

The authors declare that the research was conducted in the absence of any commercial or financial relationships that could be construed as a potential conflict of interest.

## Publisher's Note

All claims expressed in this article are solely those of the authors and do not necessarily represent those of their affiliated organizations, or those of the publisher, the editors and the reviewers. Any product that may be evaluated in this article, or claim that may be made by its manufacturer, is not guaranteed or endorsed by the publisher.
